# Electrochemical Impedance Spectroscopy in the Determination of the Dielectric Properties of Tau-441 Protein for Dielectrophoresis Response Prediction

**DOI:** 10.3390/bioengineering11070698

**Published:** 2024-07-10

**Authors:** Zuriel Shee Da En, Ervina Efzan Mhd Noor, Aminuddin Ahmed Kayani, Mohd Hazwan Hussin, Mirza Farrukh Baig

**Affiliations:** 1 Centre for Manufacturing and Environmental Sustainability (CMES), Faculty of Engineering and Technology, Multimedia University, Ayer Keroh 75450, Malaysiafarrukhbaig@mmu.edu.my (M.F.B.); 2 Functional Materials and Microsystems Research Group, School of Engineering, RMIT University, Melbourne, VIC 3001, Australia; amin.kayani@rmit.edu.au; 3 Materials Technology Research Group (MaTReC), School of Chemical Sciences, Universiti Sains Malaysia, Minden 11800, Malaysia; mhh@usm.my

**Keywords:** dielectrophoresis, Tau-441, Alzheimer’s disease, dielectric properties, electrochemical impedance spectroscopy

## Abstract

This study employs electrochemical impedance spectroscopy (EIS) to probe the behavior of Tau-441 protein, a key component implicated in Alzheimer’s disease. Through meticulous experimentation and analysis, the impedance of Tau-441 protein suspension revealed a conductivity peak value of 1.02 S/m. The study demonstrates a high level of specificity and selectivity, particularly within the challenging nanomolar concentration range. Additionally, the EIS method enabled the prediction of Tau-441 protein’s dielectrophoresis (DEP) response and the determination of the associated frequency range of 1 kHz to 1 MHz. These findings contribute to advancing our understanding of the molecular intricacies surrounding Tau-441 and hold promise for unraveling implications related to Alzheimer’s disease. This study establishes a robust foundation for future research on neurodegenerative disease and biosciences, offering valuable insights into the electrochemical dynamics of Tau-441 protein.

## 1. Introduction

Alzheimer’s disease (AD) is a progressive neurodegenerative disorder characterized by the abnormal aggregation of tau protein into neurofibrillary tangles (NFTs) and the accumulation of amyloid-beta (Aβ) plaques in the brain. Tau protein, a microtubule-associated protein, plays a crucial role in stabilizing microtubules within neurons. However, in AD, tau undergoes abnormal hyperphosphorylation, leading to its dissociation from microtubules and subsequent aggregation into NFTs, contributing to neuronal dysfunction and cognitive decline. Detecting and understanding the behavior of tau protein in AD pathology is essential for developing diagnostic tools and therapeutic strategies for this devastating disease. Tauopathies, including AD, manifest through the anomalous accumulation of tau, a hyperphosphorylated protein associated with microtubules [1]. Tau-441, a pivotal member of the Tau protein family crucial for neuronal health, is implicated in various neurodegenerative diseases. As the longest isoform with 441 amino acids, it belongs to the microtubule-associated proteins (MAPs) in the central nervous system. Six Tau isoforms, produced by alternative splicing of the MAPT gene, exist in humans. Tau-441 excessive phosphorylation and aggregation are linked to neurodegenerative diseases like Alzheimer’s disease and frontotemporal dementia [2,3,4,5,6].

Several studies have contributed to the development and application of novel biosensors for neurodegenerative diseases. Firstly, a protein-based electrochemical biosensor was devised to detect tau protein, a biomarker for such conditions, showcasing high selectivity and potential for early disease screening [7]. Secondly, a monoclonal antibody targeting tau protein labeled neurofibrillary tangles in Alzheimer’s brain tissue, shedding light on tau’s phosphorylation status [8]. Thirdly, a four-electrode electrochemical biosensor was created for rapid tau protein detection in human serum, offering promise for clinical diagnostics [9]. Lastly, a label-free biosensor was developed to specifically detect amyloid-beta oligomers, crucial in Alzheimer’s pathology, demonstrating high sensitivity and applicability in detecting both synthetic and natural amyloid-beta oligomers (AβO) [10].

Dielectrophoresis (DEP) emerges as a crucial electrokinetic phenomenon, acting on polarizable particles suspended in a liquid medium [11,12]. The movement of particles in DEP relies on the variance in polarizability between the particles and the surrounding medium [13]. When particles migrate toward the electrode edge, where the electric field gradient is high, it is termed positive DEP. Conversely, when particles move in the opposite direction to the electrode edge, the response is referred to as negative DEP [14]. During DEP, the particle inherently possesses distinct electric potentials and exhibits unique responses to varying frequencies. This phenomenon, harnessed as a novel technique, has found extensive application in biomedical laboratories [15] and microfluidics [16,17], excelling in manipulating particles for sorting, separation, and trapping within a suspended medium [18,19].

DEP force is generated through a non-uniform AC electrical field, manipulating particle movement by establishing a polarizability gradient between the particles and the surrounding medium [20]. The direction of DEP forces is voltage dependent, while frequency control allows for manipulation of the relative polarizability of cells and the medium. At a specific frequency, termed the zero-force frequency or crossover frequency, the DEP becomes zero, stabilizing the particles. This occurs when the real part of the effective polarizabilities of the particles and the surrounding medium are equal [21]. The study [22] demonstrated the successful implementation of DEP to separate and characterize amyloid-beta proteins within a microfluidic setup, showcasing controlled dissolution under specific parameters. DEP has exhibited promise in isolating amyloid proteins from nerve cells, proposing a potential approach to mitigating AD without compromising nerve function.

The effectiveness of DEP, especially for small particles like proteins, hinges on the polarization of these particles, intricately tied to their dielectric properties. Protein DEP, often exhibiting positive DEP up to the MHz range, undergoes transitions to negative DEP at a crossover frequency in the MHz range. Understanding the dielectric properties of proteins is essential, requiring the calculation of the Clausius–Mossotti (CM) factor, specifically $Re\left[f_{CM}\left(\omega \right)\right]$, the real part of the angular frequency (ω) of the applied field. In this context, predicting or determining $Re\left[f_{CM}\left(\omega \right)\right],$ essentially the crossover frequency, becomes pivotal for anticipating the DEP response, particularly in protein manipulation. Unfortunately, knowledge of protein polarization and its relation to DEP remains limited, with no documented dielectric properties for Tau-441, a protein associated with AD. To bridge this gap, established techniques like electrochemical impedance spectroscopy (EIS) offer a promising avenue. Leveraging existing knowledge, EIS can provide valuable insights into the polarization behavior of proteins under DEP conditions, thereby facilitating a deeper understanding and successful execution of protein DEP experiments.

In this study, we investigated the dielectric properties and conductivity of Tau-441 protein using EIS. A 50 µg lyophilized human recombinant Tau-441 protein, expressed in HEK 293 cells from Sigma-Aldrich, St. Louis, MO, USA, was reconstituted and analyzed using a Gamry^®^ potentiostat 600 instrument, Warminster, PA, USA. EIS measurements were conducted across a range of frequencies from 1 kHz to 10 MHz to capture the impedance spectrum of the Tau-441 protein sample. The analysis provided valuable insights into the protein’s electrical properties and facilitated predictions regarding its DEP response, offering potential applications in microfluidic systems.

## 2. Electrochemical Impedance Spectroscopy (EIS)

EIS has been reported to be a promising tool in the prediction of DEP behavior in proteins [23]. By precisely quantifying the permittivity of proteins, EIS allows for the determination of $Re\left[f_{CM}\left(\omega \right)\right]$ at a specific frequency of interest. This technique involves measuring and plotting the impedance of an electrochemical cell or electrode against frequency, aptly termed EIS. EIS can be used to evaluate small-scale chemical mechanisms at the electrode interface and within the electrolytic solution, enabling the determination of the dielectric and electrical properties of components [24,25]. Through the derivation of Euler’s relationship and the utilization of the Maxwell–Wagner model, the frequency spectra obtained through EIS can be seamlessly translated into $Re\left[f_{CM}\left(\omega \right)\right]$ frequency spectra [23]. This transformative approach not only underscores the versatility of EIS but also holds the key to unlocking a deeper understanding of protein DEP dynamics at targeted frequencies. Hence, EIS proves instrumental in discerning the cross-over frequency of the Tau-411 protein. Leveraging Euler’s relationship, impedance is expressed as a complex resistance component, characterized by both real and imaginary parts and its phase angle, encapsulated by Equation (1) [23]:

(1) $$Z*=\frac{V(t)}{I(t)}=\frac{V_{0}sin\left(\omega t\right)}{I_{0}sin\left(\omega t+\emptyset \right)}=\left|Z\right|\frac{sin\left(\omega t\right)}{sin\left(\omega t+\emptyset \right)}$$

In this context, V represents voltage, I denotes current at time t, $V_{0}$ and $I_{0}$ are the signal’s amplitude, ∅ represents the phase shift, and |Z| signifies the magnitude of impedance.

Through manipulation of experimental parameters like applied current, temperature, and reactant composition, observable frequency shifts in impedance spectra can be achieved. Euler’s relationship, $e^{j\emptyset }=cos\emptyset +jsin\emptyset$, enables the expression of Z* as a combination of real ($Z_{R}$) and imaginary ($Z_{IM}$) parts, as follows [23]:

(2) $$Z*=\left|Z\right|e^{j\emptyset }=\left|Z\right|\left(cos\emptyset +jsin\emptyset \right)=Z_{R}+jZ_{IM}$$

The phase angle at a chosen angular frequency is a ratio of the imaginary ($Z_{IM}$) and real ($Z_{R}$) impedance components [23]:

(3) $$tan\emptyset =\frac{Z_{IM}}{Z_{R}}$$

EIS measurements, conducted across a frequency range spanning from mHz to MHz, are typically graphically represented in Nyquist or Bode plots. Dynamic variations in experimental parameters, including applied current, temperature, and reactant composition, induce observable frequency shifts in impedance spectra.

EIS not only provides a comprehensive view of these shifts but also allows for the extraction of the complex permittivity of biological materials. Armed with the knowledge of the complex permittivity of both the medium and the specific biological material under scrutiny, the DEP behavior can be anticipated. This dielectric analysis unveils the intricate interplay of permittivity and conductivity, portraying them as a unified complex permittivity (ε*) characterized by real and imaginary components [23].

(4) ε*=ε′−jε″

The real permittivity component, ε′, is inversely related to Z_IM_ while the imaginary permittivity component represents the conductance of the material and is inversely proportional to Z_R_, as shown in below Equations (5) and (6) [23]:

(5) $$\varepsilon \prime =\frac{k}{\omega }\left(\frac{1}{Z_{IM}}\right)$$

(6) $$\varepsilon \prime \prime =\frac{1}{Z_{R}}=\frac{\sigma }{{\omega \varepsilon }_{0}}$$

where k represents the cell constant, which signifies the geometric parameter of the measurement volume, σ denotes the conductivity of the particle, and ε_0_ shows the vacuum permittivity.

Referring to [26], the $Re\left[f_{CM}\left(\omega \right)\right]$ factor is articulated in relation to the impedance of the particle ($Z_{p}^{*}$) and the medium ($Z_{m}^{*}$), as presented in Equation (7):

(7) $$Re\left[f_{CM}\left(\omega \right)\right]=\left(\frac{\varepsilon_{p}^{*}-\varepsilon_{m}^{*}}{\varepsilon_{p}^{*}+2\varepsilon_{m}^{*}}\right)=Re\left(\frac{Z_{m}^{*}-Z_{p}^{*}}{Z_{m}^{*}+2Z_{p}^{*}}\right)$$

where $\varepsilon_{p}^{*}$ and $\varepsilon_{m}^{*}$ are the complex permittivity of the particle and medium, respectively.

The Maxwell–Wagner model provides an insightful depiction of the impedance in a dilute suspension of particles. This model intricately defines the equivalent complex permittivity of the mixture, incorporating the ratio of the particle volume to the detection volume (volume fraction). This relationship is precisely expressed by the following equation [23]:

(8) $$\varepsilon_{mix}^{*}=\varepsilon_{m}^{*}\frac{1+2\delta f_{CM}}{1-\delta f_{CM}}$$

where $\varepsilon_{mix}^{*}$ is the complex permittivity of the mixture and δ is the volume fraction.

## 3. Methodology

A 50 µg lyophilized human recombinant Tau-441 protein, expressed in HEK 293 cells and procured from Sigma-Aldrich, underwent reconstitution following their provided instructions. Thawing for 10 min and gentle centrifugation at 500 rpm for 5 min preceded the opening of the vial. The reconstitution involved adding 5 mL of deionized water (DIW) with a conductivity of 0.055 µS/cm to achieve the recommended concentration of 0.01 mg/mL. With a molecular mass of 45.8 kDa, the resulting reconstituted Tau-441 sample, with a concentration of 218.32 nM, served as the stock sample.

To generate subsequent concentrations, 1 mL of the stock sample was mixed with 1 mL of DIW, yielding a 109.15 nM concentration. This process continued iteratively: 1 mL from the 109.15 nM concentration was diluted with 1 mL DIW, producing a 54.575 nM concentration. The cycle was repeated, with 1 mL from the 54.575 nM concentration being diluted with 1 mL DIW to obtain a 27.29 nM concentration. Finally, 1 mL of the 27.29 nM concentration sample underwent a similar dilution process with 1 mL DIW, resulting in a 13.64 nM concentration. This meticulous dilution series ensures a range of concentrations for the Tau-441 protein, facilitating precise experimentation and analysis.

The EIS experiment encompassed six distinct concentrations of Tau-441 protein, each suspended in 1 mL of deionized water (DIW). The initial test served as a control measure, devoid of Tau-441 protein, while the subsequent five tests featured varying concentrations of Tau-441 protein. Dilution of the Tau-441 protein samples adhered to the specified concentration parameters, as outlined in Table 1. This systematic approach ensures a comprehensive examination across a spectrum of Tau-441 concentrations in the EIS experiment.

In the current EIS procedures, several precautions were implemented to mitigate potential errors arising from harmonic square-wave excitation during computational calculations. Harmonic square-wave excitation was chosen for several reasons: it is faster due to the sharper edges of square waves compared to sine waves, making signal generation simpler and less complex. Additionally, square waves contain higher frequency harmonics, which are beneficial for impedance measurements and provide better sensitivity to non-linearities, crucial for characterizing non-linear behavior in impedance analysis [27]. A dummy setting was tested before each actual sample test to provide standardized background commands, guiding the computational processes to ensure consistency and accuracy. Additionally, meticulous attention was given to the proper handling and operation of electric cells, wires, and instrument power starting, ensuring precision throughout the experiments.

**Figure 1:**
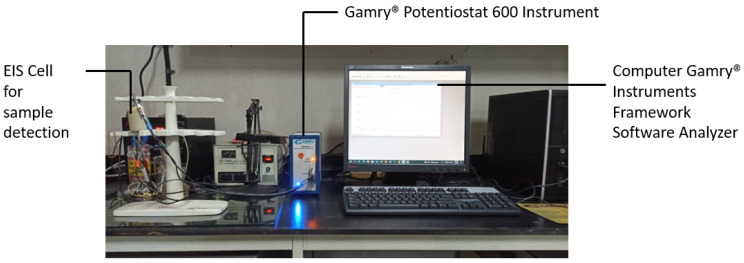
Preparation of the EIS experiment setup.

**Figure 2:**
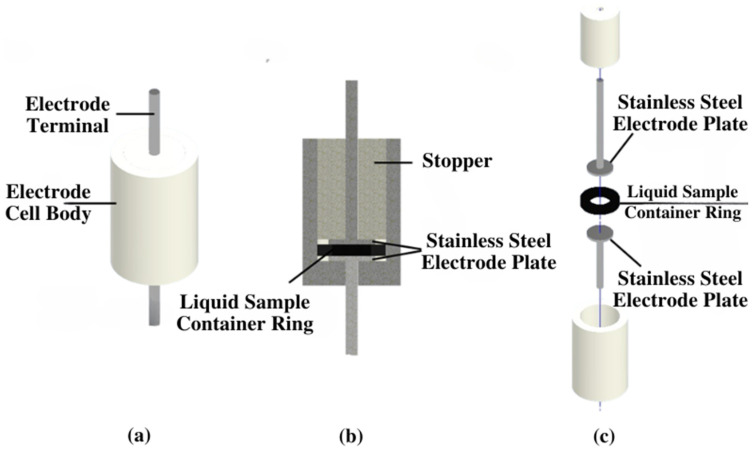
(**a**) EIS cell body illustration; (**b**) cross-section view of the cell body; (**c**) expanded view of the cell body components.

The EIS experiment was meticulously executed, as illustrated in Figure 1. Initially, a 1 mL sample of deionized water (DIW), without any containment of Tau-441 protein, was pipetted into the electrode cell and securely sealed. The Gamry^®^, Warminster, PA, USA, potentiostat 600 instrument’s wires were then connected to the electrode cell, initiating the power supply. Subsequently, within the Gamry^®^ instruments framework version 7.10 software, the settings were configured to AC 10 mV, spanning a frequency range from 0.001 Hz to 100 MHz, and the EIS analysis commenced. Following the analysis, the generated data and results were presented in Nyquist and Bode plot graphs using the Gamry^®^ instruments framework software. These valuable outcomes were recorded, and the potentiostat was powered down. The electrode cell was carefully extracted, its contents were removed, and it was thoroughly cleaned with DIW. The subsequent EIS experiments were conducted sequentially for Tau-441 sample concentrations of 218.34 nM, 109.15 nM, 54.58 nM, 27.29 nM, and 13.64 nM. Each concentration underwent the same detailed procedure for analysis, generating results data, and graphs. The cell is a bipolar cell with two terminal electrode plates. Figure 2 illustrates the EIS cell body, including a cross-section view and an expanded view of the cell body components. The dimensions of the electrode cell components are provided in Table 2.

This EIS analysis unveiled the impedance values of Tau-441 protein, discerned through adept fitting into Randles’ equivalent circuit model, resulting in the generation of Nyquist plots. After obtaining impedance data, the conductivity of the Tau-441 protein suspension was calculated using Equation (9). Expanding on the insights gained, the EIS analysis was instrumental in generating a Bode plot, offering a predictive lens into the DEP response of Tau-441. This comprehensive approach not only provides crucial information about the protein’s impedance and conductivity but also offers valuable foresight into its dynamic response to dielectrophoretic forces.

(9) $$Conductivity,\sigma =\frac{Thickness\;of\;electrode\;cell}{R_{u}\times Cross\;section\;area\;of\;the\;electrode\;cell}$$

where $R_{u}$ represents the resistance of the solution.

## 4. Results and Discussion

**Figure 3:**
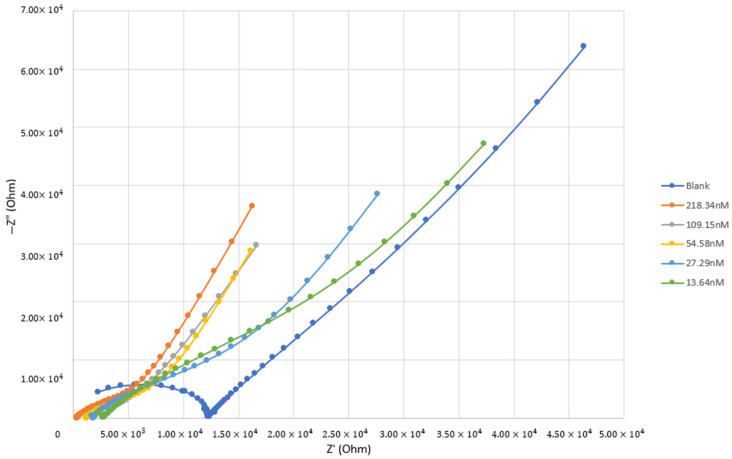
Overlays of the imaginary component of the impedance (Z_imag_) plotted against the real component of impedance (Z_real_).

**Figure 4:**
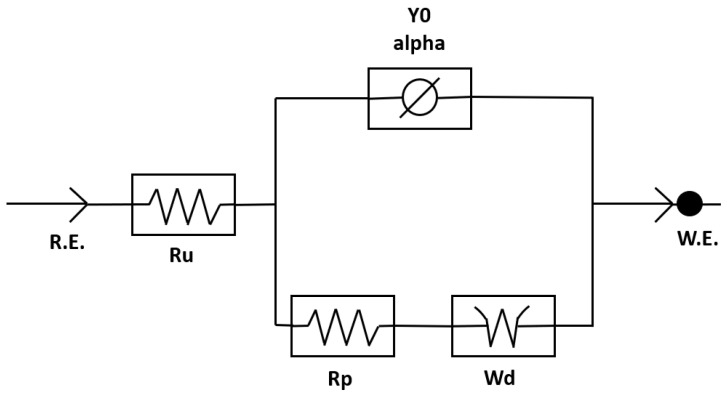
Impedance spectra fitted into a Randles’ equivalent circuit model.

EIS was used to understand how the Tau-441 protein behaves under certain electrical conditions, kind of like predicting its reactions. By measuring how the protein responds to the electrical field at a specific frequency, the Clausius–Mossotti factor, $Re\left[f_{CM}\left(\omega \right)\right]$, was calculated, providing insights into the protein’s behavior. When looking at how the impedance changes with different amounts of Tau-441 protein, a clear trend was observed—it went up in a straight line as the protein concentration increased. All of these measurements for different concentrations are shown together on a graph called a Nyquist plot in Figure 3. To dive deeper into Tau-441, a model called Randles’ equivalent circuit was used in Figure 4. This model helped to understand the connection between Tau-441’s behavior and its ability to allow electrical flow. The EIS analysis not only detected Tau-441 protein impedance but also provided parameters like resistance and other elements, giving insights into how the protein reacts in different scenarios.

Analyzing the Nyquist plot results depicted in Figure 3 involved measurements across six Tau-441 samples with varying concentrations. Starting at 218.34 nM, subsequent concentrations were prepared through sequential dilution to 109.15 nM, 54.58 nM, 27.29 nM, and 13.64 nM. The Nyquist plot effectively captured the detection of Tau-441 samples within the nanomoles (nM) range, aligning well with established patterns in the literature [28]. The outcomes revealed a notable correlation: as the Tau-441 concentration increased, resistance decreased. This phenomenon is attributed to limited electron transfer processes linked to low solution concentration and diffusion in the electrochemical process, in accordance with insights from the literature [27]. To delve into the dielectric properties of Tau-441 protein using the EIS method, the protein’s conductivity was calculated using Equation (9). Taking into account the dimensions of the electrode cell (thickness: 4.75 × 10^−3^ m; cross-section area: 1.97 × 10^−4^ m) and analyzing $R_{u}$ from the EIS Gamry^®^ instruments framework version 7.10 software, the impedance components and derived conductivity of Tau-441 protein suspension were tabulated in Table 3. This detailed analysis provides a comprehensive understanding of how Tau-441 behaves across different concentrations, shedding light on its electrochemical characteristics.

**Figure 5:**
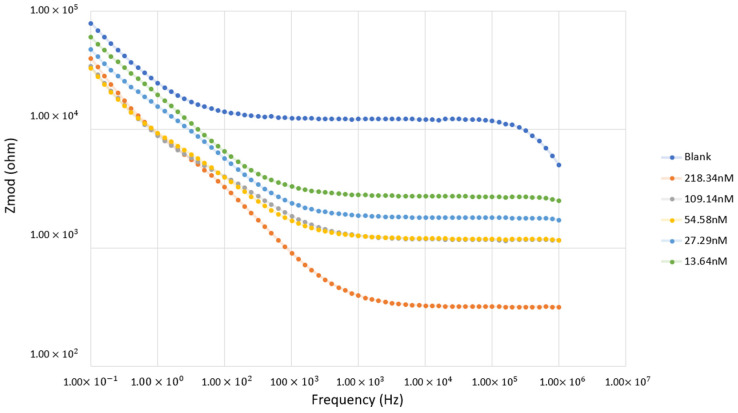
Overlays of the Bode plot of the Tau-441 protein sample.

To enhance our understanding of the DEP response of Tau-441 protein and analyze the $Re\left[f_{CM}\left(\omega \right)\right]$, factor, the investigation extended through to EIS analysis. The obtained data were then translated into a Bode plot, illustrating the modulus of the impedance (Z_real_ and Z_imag_) against frequency. Figure 5 presents the insightful Bode plot, starting from the lowest concentration of 13.64 nM to 218.34 nM Tau-441 protein. The analysis of this plot reveals a positive dielectrophoretic (DEP) response manifested by the Tau-441 protein. Based on Equation (7), wherein the Clausius–Mossotti factor is replaced via derivation with an impedance model, the Bode plot analysis indicates its region position above the positive impedance (Z_mod_) values. Hence, inferring a positive Clausius–Mossotti factor, the analysis suggests a positive DEP response for the Tau-441 protein within the optimal frequency range of 1 kHz to 1 MHz. This observation underscores the protein’s propensity for effective manipulation via dielectrophoresis within this frequency spectrum.

The EIS analysis of Tau-441 protein demonstrated a successful and highly specific detection and examination of its impedance within the concentration range of nM. Future improvements should consider factors such as variations in medium concentration. For more defined semi-circle Nyquist plots, as observed in polymer-based Tau protein detection [28], reducing the concentration to picomoles (pM) is recommended. The EIS method not only provided insights into the Tau-441 protein’s conductivity through impedance components but also successfully predicted its DEP response and determined the DEP frequency range. This comprehensive analysis, including the prediction of Tau-441 DEP behavior and the determination of dielectric properties like conductivity, holds promise for exploring implications related to Alzheimer’s disease. The high specificity achieved in the nanomolar range enhances the potential of EIS in advancing our understanding of Tau-441 protein dynamics.

Understanding the dielectric properties and conductivity of Tau-441 protein paves the way for designing effective methods for protein detection and the diagnosis of Alzheimer’s disease and offers potential therapeutic avenues for targeting Tau protein. This valuable information also enriches proteomics research, refining protein modeling for Tau-441 and enhancing our understanding of its behavior and function. These insights catalyze advancements in molecular biology and drug discovery for Alzheimer’s disease.

## 5. Conclusions

This study employed electrochemical impedance spectroscopy (EIS) to comprehensively investigate the behavior of Tau-441 protein, a crucial player in Alzheimer’s disease. Through meticulous experimentation and analysis, the impedance of Tau-441 protein was successfully detected and analyzed. Notably, its conductivity value reached a peak at an impressive 1.02 S/m. This achievement was realized with remarkable specificity and selectivity, particularly within the challenging nanomolar concentration range. To enhance the precision of our results, future refinements should consider reducing the medium concentration to picomoles, as suggested by the observed semi-circle Nyquist plots in analogous studies. Additionally, attention to factors such as medium diffusion, as indicated by the presence of a Warburg resistor in the Randles’ equivalent circuit model, further refines our understanding of the experimental conditions. Crucially, the present EIS analysis not only determined the conductivity of Tau-441 protein suspension but also successfully predicted its dielectrophoresis (DEP) response and the associated frequency range. These findings offer valuable insights into the electrochemical dynamics of Tau-441, laying the groundwork for potential implications in Alzheimer’s disease.

## Figures and Tables

**Table 1:** Molarity concentration of Tau-441 samples for the EIS experiment.

Test No.	Molarity	Volume
1	(DIW sample with no Tau-441 protein)	1 mL
2	218.34 nM	1 mL
3	109.15 nM	1 mL
4	54.58 nM	1 mL
5	27.29 nM	1 mL
6	13.64 nM	1 mL

**Table 2:** Dimensions of the electrode cell components.

Components	Dimensions (mm)
Ring Thickness	4.75
Ring Inner Diameter (ID)	15.82
Ring Outer Diameter (OD)	28
Stainless Steel Plate Diameter	19
Stainless Steel Plate Thickness	2
Stainless Steel Terminal Diameter	5
Stainless Steel Terminal Length	75 and 45
Stopper	OD: 28, ID: 5.1, H: 41.25
Cell Body	OD: 40, height: 60, ID: 28.1, height: 50, hole: 5.1

**Table 3:** Tau-441 impedance components obtained from the EIS analysis and the derived conductivity value.

Concentration	$R_{u}$ (Ω)	Y0 (F)	Alpha (F)	Wd (Ω)	Rp (Ω)	Conductivity (S/m)
Blank	1.228 × 10^−3^	29.36 × 10^−12^	1.0	23.53 × 10^−6^	11.39 × 10^3^	2.12 × 10^−3^
218.34 nM	313.2	12.43 × 10^−6^	719.9 × 10^−3^	28.53 × 10^−6^	3.785 × 10^3^	6.37 × 10^−3^
109.15 nM	1.153 × 10^3^	13.68 × 10^−6^	653.9 × 10^−3^	33.99 × 10^−6^	2.925 × 10^3^	8.24 × 10^−3^
54.58 nM	1.178 × 10^3^	13.21 × 10^−6^	695.9 × 10^−3^	34.94 × 10^−6^	4.038 × 10^3^	5.97 × 10^−3^
27.29 nM	1.787 × 10^3^	7.768 × 10^−6^	747.9 × 10^−3^	24.28 × 10^−6^	8.381 × 10^3^	2.88 × 10^−3^
13.64 nM	2.682 × 10^3^	1.898 × 10^−6^	869.8 × 10^−3^	19.89 × 10^−6^	23.69	1.02

## Data Availability

The raw data supporting the conclusions of this article will be made available by the authors on request.
